# Antioxidants cause rapid expansion of human adipose-derived mesenchymal stem cells via CDK and CDK inhibitor regulation

**DOI:** 10.1186/1423-0127-20-53

**Published:** 2013-08-01

**Authors:** Li-Yi Sun, Cheng-Yoong Pang, Dian-Kun Li, Chia-Hsin Liao, Wei-Chao Huang, Chao-Chuan Wu, Yi-Yo Chou, Wei Wu Li, Shin-Yuan Chen, Hwan-Wun Liu, Yao-Jen Chang, Ching-Feng Cheng

**Affiliations:** 1 Department of Medical Research, Buddhist Tzu Chi General Hospital, Hualien, No. 707, Sec. 3, Zhongyang Rd., Hualien City 970, Hualien, Taiwan; 2 Institute of Medical Sciences, Tzu Chi University, Hualien, Taiwan; 3 Department of Hematology and Oncology, Taichung Tzu Chi General Hospital, Taichung, Taiwan; 4 Department of Plastic Surgery, Taipei Tzu Chi General Hospital, Taipei, Taiwan; 5 Department of Surgery, Buddhist Tzu Chi General Hospital Taipei Branch, Taipei, No. 289, Jianguo Rd., Xindian Dist., New Taipei City 231, Taiwan; 6 Department of Neurosurgery, Buddhist Tzu Chi General Hospital, Hualien, Taiwan; 7 Department of Occupational Medicine, Buddhist Tzu Chi General Hospital, Hualien, Taiwan; 8 Institute of Biomedical Sciences, Academia Sinica, Taipei, Taiwan

**Keywords:** Adipose-derived mesenchymal stem cells, Antioxidant, Fibroblast growth factor-2, Hypoxia

## Abstract

**Background:**

Antioxidants have been shown to enhance the proliferation of adipose-derived mesenchymal stem cells (ADMSCs) *in vitro*, although the detailed mechanism(s) and potential side effects are not fully understood.

In this study, human ADMSCs cultured in ImF-A medium supplemented with antioxidants (*N*-acetyl-l-cysteine and ascorbic acid-2-phosphate) and fibroblast growth factor 2 (FGF-2) were compared with ADMSCs cultured with FGF-2 alone (ImF) or with FGF-2 under 5% pO_2_ conditions (ImF-H).

**Results:**

During log-phase growth, exposure to ImF-A resulted in a higher percentage of ADMSCs in the S phase of the cell cycle and a smaller percentage in G0/G1 phase. This resulted in a significantly reduced cell-doubling time and increased number of cells in the antioxidant-supplemented cultures compared with those supplemented with FGF-2 alone, an approximately 225% higher cell density after 7 days. Western blotting showed that the levels of the CDK inhibitors p21 and p27 decreased after ImF-A treatment, whereas CDK2, CDK4, and CDC2 levels clearly increased. In addition, ImF-A resulted in significant reduction in the expression of CD29, CD90, and CD105, whereas relative telomere length, osteogenesis, adipogenesis, and chondrogenesis were enhanced. The results were similar for ADMSCs treated with antioxidants and those under hypoxic conditions.

**Conclusion:**

Antioxidant treatment promotes entry of ADMSCs into the S phase by suppressing cyclin-dependent kinase inhibitors and results in rapid cell proliferation similar to that observed under hypoxic conditions.

## Background

Human mesenchymal stem cells (MSCs) are emerging as powerful tools in regenerative medicine. Clinical applications using MSCs have recently been reported for treating acute diseases, such as acute myocardial infarction [1], hemorrhagic stroke [2], and acute soft-tissue injuries [3]. To reduce patient waiting time, culture conditions are needed for rapid and reproducible *in vitro* expansion of MSCs. Current methods under development for obtaining large quantities of high-quality MSCs include the use of bioreactors [4-7], different types of serum [8,9], platelet lysates [10], and growth factors [11].

Recently, hypoxia (1%–5% pO_2_) was also shown to enhance cell growth [12,13] and cell homing ability [14,15]. The osteogenic and chondrogenic potentials of bone marrow mesenchymal stem cells (BMMSCs) exposed to hypoxic conditions were increased relative to those cultured under normoxic conditions (approximately 21% pO_2_), possibly via a hypoxic effect on the regulation of the cell cycle [13]. Thus, it is possible that high oxygen environments, that increase the levels of reactive oxygen species (ROS) through cellular respiration are not suitable for large-scale production of high-quality MSCs in a stir system. However, some studies have shown that a culture medium with low calcium levels and supplemented with antioxidants can accelerate the growth and prolong the lifespan of adipose-derived mesenchymal stem cells (ADMSCs) under normoxic conditions [16,17]. According to these studies, the effects of antioxidants on cell expansion of ADMSCs are similar to those of hypoxia on BMMSCs. However, mechanism(s) by which antioxidants exert these effects on ADMSCs remains unclear.

ADMSCs have recently been identified as more powerful tools for regenerative medicine than BMMSCs because they can be expanded more rapidly [18,19], they secrete cytokines and growth factors [20,21], and they can be obtained in large numbers from liposuction aspirates [16,22-24]. In addition, it has been suggested that fibroblast growth factor 2 (FGF-2) is the most effective factor for promoting the growth of BMMSCs *in vitro*[11] and that it enhances the osteogenic and chondrogenic potential of these cells [25,26]. We therefore hypothesized that, compared with hypoxia, a combination of antioxidants and FGF-2 may be a more rapid and easy method to control the culture of ADMSCs *in vitro*. We also speculated that antioxidants may act through the same mechanism(s) as hypoxia promotes MSC proliferation. Thus, in the present study, we examined the effects of antioxidants and FGF-2 on proliferation, cell cycle regulation, cell-surface antigens, stemness-related gene expression, and differentiation potential in comparison with a combination of either normoxia and FGF-2 or hypoxia and FGF-2.

## Methods

### Isolation and maintenance of human ADMSCs

This study was approved by the Buddhist Tzu Chi General Hospital Internal Review Board (IRB100-102). Stromal-vascular fraction (SVF) cells were isolated using a method modified from that described by Griesche and colleagues [27]. Human adipose tissues were diluted 2-fold with phosphate-buffered saline (PBS, GIBCO-Invitrogen), and collagenase type I (final concentration: 0.4 mg/mL; Sigma) was added for enzymatic digestion in a hybridization oven (37°C, 30° angle, 15 rpm, 45 min; RH-800D, YIHDER). Digested adipose tissues were centrifuged at 400 × *g* for 10 min to generate the SVF pellet, which was resuspended in PBS and filtered through a 70-μm nylon mesh (Becton Dickinson) to isolate SVF for subsequent ADMSCs culture. Experiments were conducted using ADMSCs at passages 2 to 5. To maintain and expand ADMSCs populations, the cells were cultured in MSC maintenance medium containing Iscove’s modified Dulbecco’s medium (IMDM; GIBCO-Invitrogen) and 10% fetal bovine serum (FBS, MSC-Qualified, GIBCO-Invitrogen) with 10 ng/mL FGF-2 (R&D Systems) as described [6,7].

### Culture of ADMSCs with antioxidants and FGF-2 under normoxic or hypoxic conditions

**Figure 1:**
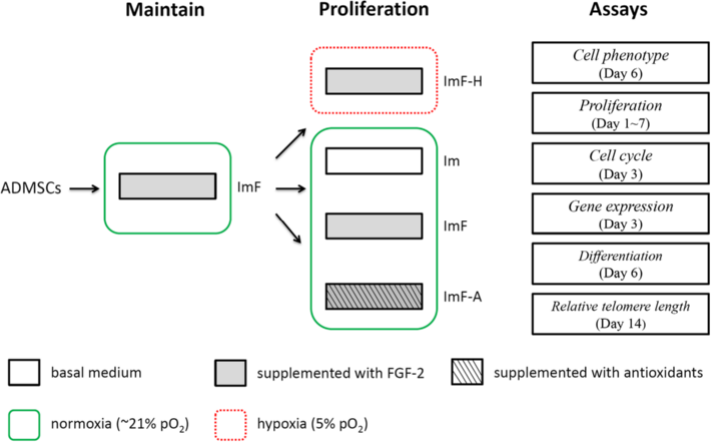
Overview of ADMSCs culture conditions and assay design.

Figure 1 shows a schematic diagram of the four culture processes. For the experiments described herein, ADMSCs were seeded at an initial cell density of 3,000 cells/cm^2^ in tissue culture flasks or 6-well plates (Becton Dickinson). The normoxic environment was maintained at 37°C in a humidified 5% pCO_2_ incubator (Forma Series II Model 3110, Thermo), and the hypoxic environment was maintained at 37°C in a humidified incubator (MCO-18 M, Sanyo) containing 5% pO_2_ and 5% pCO_2_. The experimental group (ImF-A) was cultured in IMDM supplemented with 10% FBS, 10 ng/mL FGF-2, 2 mM *N*-acetyl-l-cysteine (NAC, Sigma), and 0.2 mM L-ascorbic acid-2-phosphate (AsA2P, Sigma) in the normoxic environment. The positive control group was cultured in IMDM supplemented with 10% FBS and 10 ng/mL FGF-2 in the hypoxic environment (ImF-H). The negative controls were cultured in the normoxic environment with (ImF) or without (Im) 10 ng/mL FGF-2. Starting on day 3, the media were completely changed every 3 days for all groups.

### Cell proliferation and viability assays

For cell proliferation and viability assays, cells were detached by trypsinization (GIBCO-Invitrogen) and use of a cell scraper (Becton Dickinson). They were then counted in triplicate using a cell counter (Vi-CELL AS, Beckman Coulter). Cell viability was measured using 0.4% (w/v) trypan-blue (GIBCO-Invitrogen) exclusion and software (Beckman Coulter) set to the parameter values recommended for ADMSCs (100 images, 10–30 μm, 75% spot brightness, 5% spot area). Doubling time was calculated as = time/(3.32[log_10_N_t_ - log_10_N_0_]), where N_t_ and N_0_ are the final and initial cell densities, respectively.

### Cell population and cell-surface antigen analysis

Antibodies against human CD13, CD14, CD29, CD34, CD44, CD45, CD73, CD90, CD105, β_2_ microglobulin (B2M), and HLA-DR were purchased from Becton Dickinson. For the characterization of cell-surface antigen phenotype, the ADMSCs were cultured in 75-cm^2^ tissue culture flasks (Becton Dickinson) at an initial density of 3,000 cells/cm^2^ for 6 days. Cells were harvested and stained with an antibody coupled to fluorescein isothiocyanate or R-phycoerythrin, and then analyzed by flow cytometry (FACSCalibur, Becton Dickinson). ADMSC populations were assessed by their forward (size) and side (granularity) scattering properties deduced by flow cytometry.

### Cell cycle analysis

For cell cycle analysis, 75-cm^2^ tissue culture flasks seeded with ADMSCs (initial density, 3000 cells/cm^2^) were placed in the aforementioned four culture environments for 3 days. Flasks (n = 3) were removed from the incubator to detach and harvest the cells for cell cycle phase identification as reported [6]. The propidium iodide (Sigma) staining and percentages of cells in each phase of the cell cycle were analyzed by flow cytometry and MultiCycle software (Phoenix Flow Systems).

### Analyses of gene expression and telomere length

Isolation of total RNA, cDNA synthesis, and amplification reactions were carried out as described [28]. Total RNA was isolated from cells using the RNeasy kit (Qiagen), and cDNA was synthesized from 10 μg of total RNA using the Advantage RT-for-PCR kit (Clontech). The resulting cDNA was subjected to PCR amplification using the gene-specific primers listed in Table 1 to identify how the different cell conditions affected the expression levels of *p21* (cyclin-dependent kinase inhibitor 1A), *p27* (cyclin-dependent kinase inhibitor 1B), *Nanog*, *Oct4* (octamer-binding transcription factor 4), *SOX2* (sex-determining region Y-box 2), *CXCR4* (C-X-C chemokine receptor type 4), *HIF1A* (hypoxia inducible factor 1, alpha subunit), *VEGF* (vascular endothelial growth factor), *TGF-β1* (transforming growth factor beta 1), *cbfa1* (core-binding factor subunit alpha-1), *OC* (osteocalcin), *PPARr* (proliferator-activated receptor γ), *C/EBPα* (CCAAT⁄enhancer-binding protein α), *ACAN* (aggrecan), *COL2A1* (collagen type II, alpha 1), and *β-actin*. Gene expression levels were normalized to that of the *β-actin* gene, which served as the internal control. The relative telomere lengths of ADMSCs (from passage 2 to passage 5) after 14 days under the four conditions were determined using the telomere/single-gene ratio detected using the method described by Cawthon [29]. DNA was isolated using the QIAamp DNA mini kit (Qiagen). The gene-specific primers are listed in Table 1. The amplified genes included *36B4* and *Telomeres*. Quantitative PCR and product detection were performed using the Fast Start Essential DNA Green Master (Roche) and the PikoReal Real-Time PCR System (Thermo Scientific), and analyzed using PikoReal 2.0 software (Thermo Scientific).

**Table 1:** Primers used for real-time PCR

**Genes**	**Primer sequences**	**Product size (bp)**
*β-actin*	S: 5′-CGCCAACCGCGAGAAGAT-3′	168
	A: 5′-CGTCACCGGAGTCCATCA-3′	
*P21*	S: 5′-CCGAAGTCAGTTCCTTGTGG-3′	112
	A: 5′-CATGGGTTCTGACGGACAT-3′	
*P27*	S: 5′-TTTGACTTGCATGAAGAGAAGC-3′	84
	A: 5′-AGCTGTCTCTGAAAGGGACATT-3′	
*Nanog*	S: 5′-AATACCTCAGCCTCCAGCAGAT-3′	148
	A: 5′-TGCGTCACACCATTGCTATTCTT-3′	
*Oct4*	S: 5′-CTTGCTGCAGAAGTGGGTGGAGGAA-3′	187
	A: 5′-CTGCAGTGTGGGTTTCGGGCA-3′	
*SOX9*	S: 5′-AGACCAGTACCCGCATCT-3′	108
	A: 5′-CGCTCCGCCTCCTCCAC-3′	
*CXCR4*	S: 5′-CGTGGAACGTTTTTCCTGTT-3′	129
	A: 5′-TGTAGGTGCTGAAATCAACCC-3′	
*HIF1A*	S: 5′-CAGCAACTTGAGGAAGTACC-3′	139
	A: 5′-CAGGGTCAGCACTACTTCG-3′	
*VEGF*	S: 5′-ACGATCGATACAGAAACCACG-3′	105
	A: 5′-CTCTGCGCAGAGTCTCCTCT-3′	
*TGF-β1*	S: 5′-TAAATACAGCCCCCATGGCA-3′	243
	A: 5′-GTCCTGGCCCTGTACAACC-3′	
*Cbfa1*	S: 5′-TGGCAGCACGCTATTAAATC-3′	103
	A: 5′-TCTGCCGCTAGAATTCAAAA-3′	
*OC*	S: 5′-CAAAGTCTAACTAGGGATACC-3′	150
	A: 5′-AGAGATGAGTCTGTCCTG-3′	
*PPARγ*	S: 5′-TTGCTGTCATTATTCTCAGTGGA-3′	124
	A: 5′-GAGGACTCAGGGTGGTTCAG-3′	
*CEBPα*	S: 5′-GACATCAGCGCCTACATCG-3′	70
	A: 5′-GGCTGTGCTGGAACAGGT-3′	
*ACAN*	S: 5′-TACACTGGCGAGCACTGTAAC-3′	71
	A: 5′- CAGTGGCCCTGGTACTTGTT-3′	
*COL2A1*	S: 5′- GAATAGCACCATTGTGTAGGAC-3′	97
	A: 5′- AATGCCCCCTGAGTGAC-3′	
*36B4*	S: 5′-CAGCAAGTGGGAAGGTGTAATCC-3′	75
	A: 5′-CCCATTCTATCATCAACGGGTACAA-3′	
*Telomeres*	S: 5′-GGTTTTTGAGGGTGAGGGTGAGGGTGAGGGTGAGGGT-3′	>76
	A: 5′-TCCCGACTATCCCTATCCCTATCCCTATCCCTATCCCTA-3′	

*S*: sense, *A*: antisense.

### Western blot analysis

These procedures were modified from that described by Chiu et al. [30]. Cells were detached using a cell scraper and then resuspended in lysis buffer (50 mM Tris-HCl, pH 7.5, 0.5 M NaCl, 5 mM MgCl_2_, 0.5% Nonidet P-40, 1 mM phenylmethylsulfonyl fluoride, 1 μg/mL pepstatin, and 50 μg/mL leupeptin) on ice and centrifuged at 13,000 × *g* at 4°C for 30 min. Protein (20 μg) was collected, subjected to 10% SDS-PAGE, and transferred to a polyvinylidene difluoride membrane blocked with 5% non-fat milk in Tris-buffered saline containing 0.1% Tween 20 for 1 h at room temperature. The various membranes were probed with appropriate dilutions of the following primary antibodies at 4°C overnight: cyclin A2, cyclin D1, cyclin D3, p21, p27, cyclin-dependent kinase (CDK) 2, CDK4, CDK6, and cell division control protein 2 (CDC2) (Cell Signaling Technology). After the membranes were washed three times with Tris-buffered saline (0.1% Tween 20), they were incubated with a horseradish peroxidase–conjugated secondary antibody (anti-mouse or anti-rabbit, Cell Signaling Technology) for 1 h at room temperature. All resolved protein bands were analyzed with ImageJ 1.40 g software (NIH).

### In vitro differentiation analysis

Osteogenesis and adipogenesis were induced using established protocols [6]. ADMSCs recovered from Im, ImF, ImF-H, and ImF-A at day 6 were re-plated onto 35-mm dishes (Becton Dickinson) at 10,000 cells/cm^2^, and osteogenic differentiation potential was evaluated using alkaline phosphatase staining and *cbfa1* and *OC* expression at day 14. The adipogenic differentiation potential of ADMSCs was evaluated using Oil red O staining and *PPARγ* and *C/EBPα* expression at day 7. Methods for alkaline phosphatase staining and Oil red O staining were previously reported [6,7].

Chondrogenesis was induced using chondrogenic medium and the micromass culture method [17] consisting of 1 × 10^5^ cells/10 μl in 24-well plates for 2 hours and then incubated in chondrogenic medium. Chondrogenic medium consisted of high-glucose DMEM (Sigma), supplemented with ITS + premix (Sigma), 10 ng/mL transforming growth factor β1 (R&D), 100 μg/mL sodium pyruvate (Sigma), 50 μg/ml ascorbic acid (Sigma), and 0.1 μM dexamethasone (Sigma). The chondrogenic differentiation potential was evaluated by using Alcian Blue (Sigma) staining of sulfated proteoglycan-rich matrix (blue colour) and *ACAN* and *COL2A1* expression at day 14.

### Statistical analyses

Statistical analyses for cell proliferation, cell population characterization, cell cycle phase analysis, and gene expression in each group were performed using the Microsoft Excel data analysis program t-test, and *P* values < 0.05 were considered statistically significant. Experiments were performed at least twice, and each value represents the mean ± SD (n = 3 for replicates).

## Results

### Effects of antioxidants and FGF-2 on ADMSC proliferation

**Figure 2:**
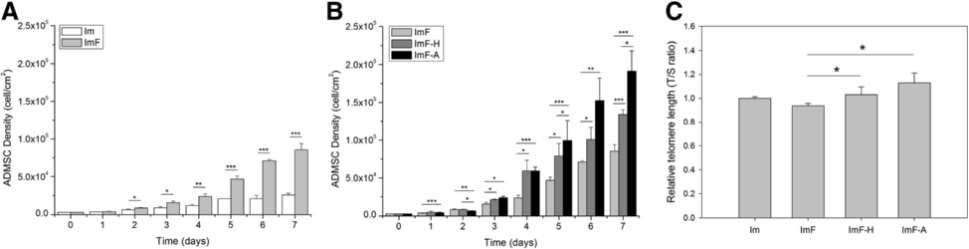
**Effects of FGF-2 and antioxidants on ADMSCs proliferation under normoxic or hypoxic conditions. (A)** Cell density in the presence (ImF) or absence (Im) of FGF-2. **(B)** Cell density in the presence of FGF-2 (ImF), FGF-2 with antioxidants (ImF-A), or FGF-2 under hypoxic conditions (ImF-H). **(C)** Relative telomere length of ADMSCs after 14 days under the four culture conditions as assessed by quantitative PCR. Medium was changed every 3 days, starting on the third day. Each bar represents the mean value ± SD (n = 3). * *P* < 0.05. ** *P* < 0.01. *** *P* < 0.005.

As shown in Figure 2A and Table 2, cell density significantly differed between ADMSCs grown in Im and those grown in ImF after 2 days. There was an approximately 1.2-fold decrease in the log-phase doubling time in the ImF cultures (Table 2). Similarly, there was an approximately 3.3-fold increase in the number of cells obtained from the ImF cultures after 7 days. As shown in Figure 2B and Table 2, the doubling time of ADMSCs was decreased relative to ImF culture after ImF-A or ImF-H culture. In addition, there were significant fold differences in cell densities and log-phase doubling time between ImF-A group and the respective control groups (Im and ImF), with approximately 225%–740% higher cell densities after 7 days in culture.

**Table 2:** Effects of different cell culturing methods on growth kinetic parameters

**Cell type**	**Conditions**	**Fold increase in cell density (7 days)**	^**# **^**Doubling time (h)**
ADMSC	Im	8.6	28.6 ± 4.9
	ImF	^a^ 28.4	22.6 ± 3.4
	ImF-H	^a,b^ 44.7	17.8 ± 6.1
	ImF-A	^a,b,c^ 63.8	^a,b^ 13.1 ± 1.0

*P* < 0.05, ^a^vs. Im, ^b^vs. ImF, ^c^vs. ImF-H.

^#^Doubling time represents the minimal doubling time during log phase.

To investigate the effect of the combination of antioxidants and FGF-2 on cellular aging, relative telomere lengths of ADMSCs after 14 days under the four conditions were determined from the telomere/single gene ratio. As shown in Figure 2C, telomeres were relatively longer in ADMSCs in the ImF-A and ImF-H groups relative to the ImF group.

### Effects of antioxidants and FGF-2 on cell cycle regulation

**Figure 3:**
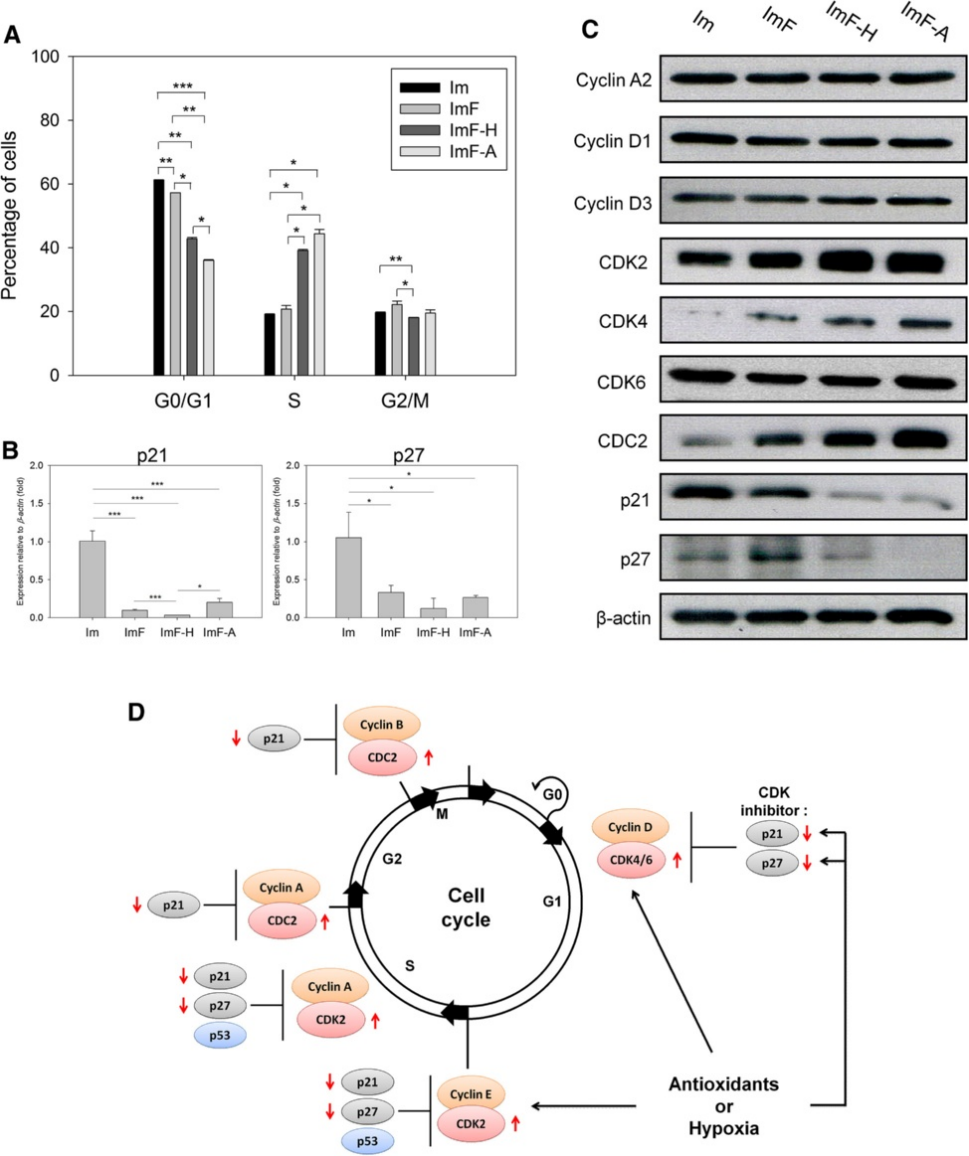
**Effects of FGF-2, antioxidants, and hypoxia on the cell cycle profiles of ADMSCs. (A)** The percentage of cells in each phase of the cell cycle at day 3. **(B)** Relative levels of p21 and p27 mRNAs in ADMSCs from the various culture conditions. Expression of *β-actin* served as the internal control. Expression values were normalized to the corresponding values measured for cells cultured under the Im condition. **(C)** Western blot analysis showed that CDK4 increased, but p21 and p27 decreased, in cells cultured under ImF-H or ImF-A conditions. **(D)** Schematic diagram of antioxidant effects on cell cycle progression. The bars represent the mean value ± SD (n = 3). * *P* < 0.05. ** *P* < 0.01. *** *P* < 0.005.

To further investigate the mechanism(s) underlying the stimulation of proliferation by antioxidants, we used flow cytometry analysis in conjunction with propidium iodide staining to determine the proportion of ADMSCs in various cell cycle stages (Figure 3A). Significant differences were observed between ADMSCs cultured in ImF-A and ImF-H relative to the control groups (Im and ImF) at day 3 (during log-phase growth), at which time exposure to both antioxidants and hypoxia resulted in a greater percentage of cells in the S phase and a smaller percentage in the G0/G1 phase.

In addition, reverse transcription quantitative PCR (RT-qPCR) analysis (Figure 3B) showed that expression of the CDK inhibitors *p21* and *p27* decreased after FGF-2 treatment. Western blot analysis (Figure 3C) showed that levels of the CDK inhibitors p21 and p27 also decreased in the antioxidant-treated cells (ImF-A), although CDK2, CDK4, and CDC2 levels clearly increased. Similar results were obtained with hypoxia-treated cells (ImF-H), although the extent of change from the control group (ImF) was smaller than that with antioxidant treatment.

### Effects of antioxidants and FGF-2 on stemness-related gene expression

**Figure 4:**
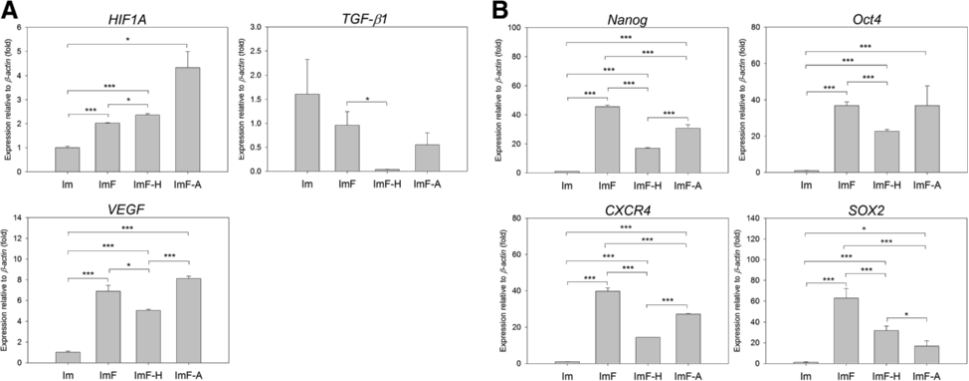
**Changes in expression of growth factor and stemness genes under the different culture conditions. (A)** Relative expression levels of two growth factor genes in ADMSCs from the four different conditions. **(B)** Relative expression levels of stemness genes in ADMSCs from the four different conditions. Expression of *β-actin* served as the internal control. The gene expression ratios were normalized to the corresponding levels measured for cells cultured under the Im condition. Each bar represents the mean value ± SD (n = 3). * *P* < 0.05. ** *P* < 0.01. *** *P* < 0.005.

RT-qPCR analysis showed that FGF-2 enhanced the expression of genes, including *Nanog*, *Oct4*, *SOX2*, *CXCR4*, *HIF1A*, and *VEGF* (Figure 4). However, ImF-A or ImF-H decreased the expression of *Nanog*, *SOX2*, *CXCR4* and *TGF-β1*. Moreover, the expression of *Nanog*, *Oct4*, *CXCR4*, *HIF1A*, and *VEGF* was significantly lower in the ImF-H group than in the ImF-A group.

### Effects of antioxidants and FGF-2 on cell morphology, cell populations, and immunophenotypes

**Figure 5:**
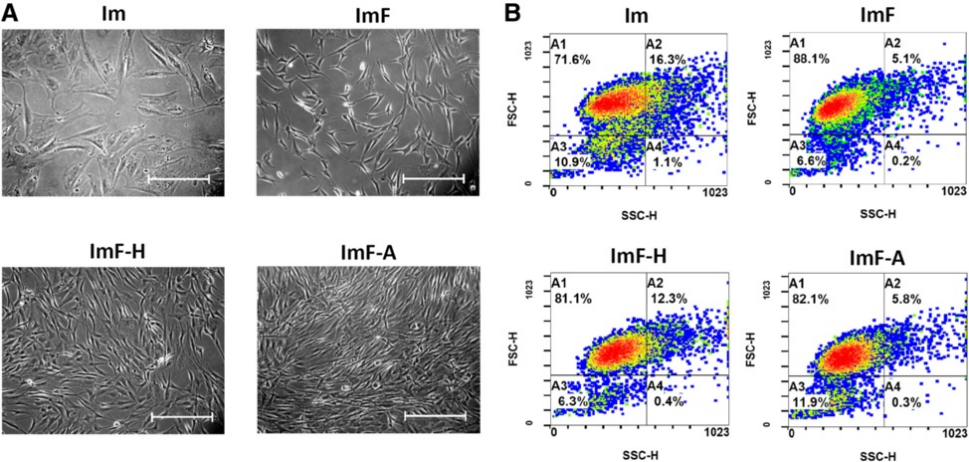
**Effects of different conditions on the morphology and cell population of ADMSCs. (A)** Photomicrographs of ADMSCs. Bar = 500 μm. **(B)** Changes in cell size and granularity of ADMSCs as characterized by forward-scatter (FS) versus side-scatter (SS) density plots derived from flow cytometry. The density plots are divided into four quadrants (A1–A4). The density threshold is 50 cells, and density levels between 1 and 5 are colored from blue (least dense) to red (most dense).

As shown in Figure 5A, when FGF-2 was added to the medium (ImF, ImF-H, ImF-A), ADMSCs maintained their spindle-shaped morphology. The size and granularity of ADMSCs under the different culture conditions were also assayed by their forward-and side-scatter properties as assessed using flow cytometry (Figure 5B). Compared with the Im group, the percentage of agranular cells (A1 quadrant) in the FGF-2 groups (ImF, ImF-H, ImF-A) increased after 6 days in culture. The percentage of granular cells (A2 quadrant) in the ImF-H group was substantially higher than that in the normoxic environment (ImF and ImF-A; approximately 20% pO_2_).

In addition, flow cytometry analysis of ADMSC immunophenotypes showed that FGF-2 treatment for 6 days resulted in significant reductions in CD105 (endoglin) expression (Table 3). However, CD29 (integrin β1, fibronectin receptor) and CD105 expression decreased more in the cells treated with ImF-A than in those treated with ImF-H for 6 days. The effects of these treatments on CD105 were further confirmed in the EdU-labeled cells experiments: Additional file 1: Figure S1 showed that FGF-2, hypoxia, and antioxidants all resulted in higher percentage of EdU-labeled ADMSCs of low CD105 expression (F4 division), but the reductions of CD105 were also found in non-proliferated cells (F1, EdU-negative cells).

**Table 3:** Effect of the four culture conditions on ADSC surface markers

**Surface marker**	**Im**	**ImF**	**ImF-H**	**ImF-A**
*CD13*	+++	+++	+++	+++
*CD14*	-	-	-	-
*CD29*	+++	+++	++	++
*CD34*	-	-	-	-
*CD44*	+++	+++	+++	+++
*CD45*	-	-	-	-
*CD73*	+++	+++	+++	+++
*CD90*	+++	+++	++	++
*CD105*	+++	++	++	+
*B2M*	+++	+++	++	++
*HLA-DR*	-+	-	-	-

-: 0 ~ 5%; -+:5 ~ 25%; +:25 ~ 50%; ++: 50 ~ 95%; +++: 95 ~ 100%.

### Effects of antioxidants and FGF-2 on the differentiation potential of ADMSCs

**Figure 6:**
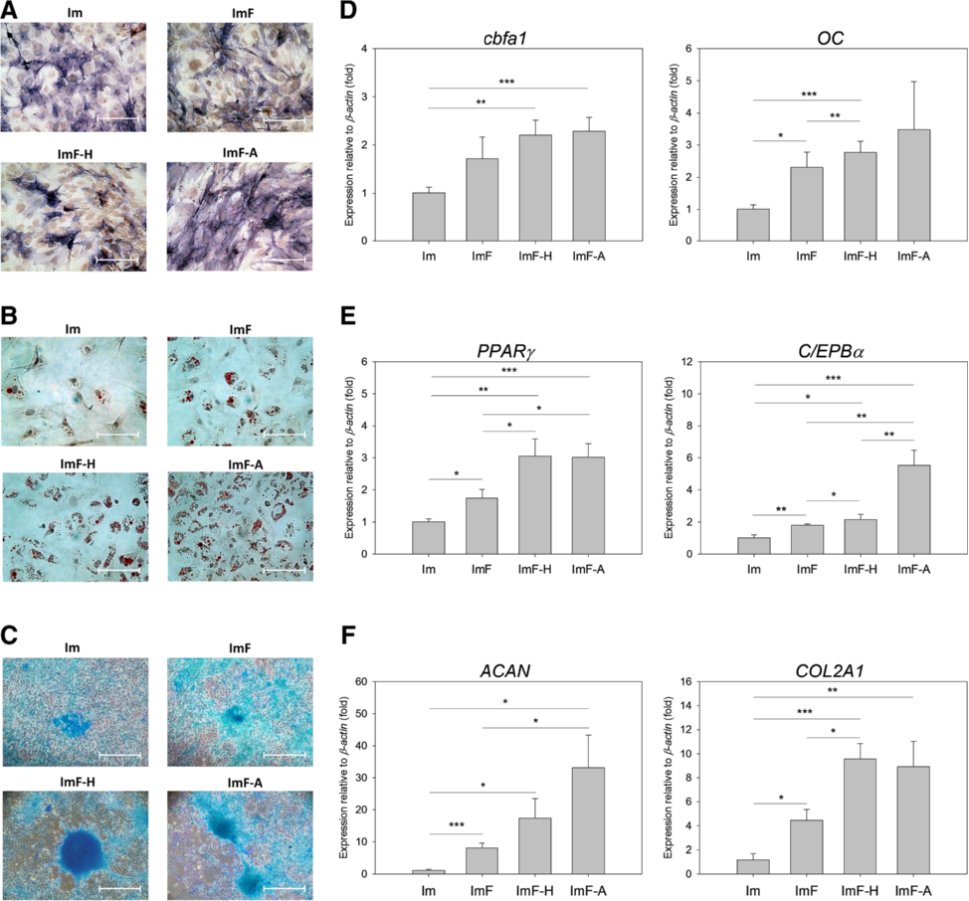
**Differentiation potential of ADMSCs after 6 days under the four culture conditions. (A)** Photomicrographs showing alkaline phosphatase activity of ADMSCs after 14 days osteo-differentiation. Bar = 500 μm. **(B)** Photomicrographs of lipid spheres in ADMSCs after 7 days adipo-differentiation. Lipid spheres were visualized by Oil Red O staining. Bar = 500 μm. **(C)** Photomicrographs of sulfated proteoglycan-rich matrix (blue color) in ADMSCs after 14 days chondro -differentiation. **(D)** Quantification of osteogenic mRNA in ADMSCs after 14 days osteo-differentiation. **(E)** Quantification of adipogenic mRNA in ADMSCs after 7 days adipo-differentiation. **(F)** Quantification of chondrogenic mRNA in ADMSCs after 7 days adipo-differentiation. Expression of *β-actin* served as the internal control. Each bar represents the mean value ± SD (n = 3). * *P* < 0.05. ** *P* < 0.01. *** *P* < 0.005.

As shown in Figure 6A-6C, ADMSCs that had undergone the different treatments could differentiate into osteoblasts, chondrocytes, or adipocytes. RT-qPCR analysis showed that osteo-differentiated ADMSCs from the ImF-A group expressed higher levels of *cbfa1* and *OC* than those from the Im group (Figure 6D), and chondro-differentiated ADMSCs from the ImF-A group expressed higher levels of *ACAN* and *COL2A1* than cells from the Im and ImF groups (Figure 6F). After antioxidant treatment, adipo-differentiated ADMSCs also expressed higher levels of *PPARγ* and *C/EPBα* than cells from the Im and ImF groups (Figure 6E), and the number of Oil Red O-positive lipid vacuoles was increased in these cells. As indicated in Figure 6D-6F, our data suggest that FGF-2 enhances the osteogenic, chondrogenic, and adipogenic differentiation potential of ADMSCs and a combination of antioxidants and FGF-2 further enhances this potential. However, the changes in expression of osteogenic, chondrogenic and adipogenic genes that were observed in the ImF-A group after 6 days exposure were similar to that observed in the ImF-H group.

## Discussion

The detailed mechanisms by which antioxidants induce cell proliferation remain unclear, although the effects of antioxidants on ADMSC growth have been examined by some investigators [16,17,31]. In this study, we observed that both hypoxia and antioxidants increased ADMSC proliferation by increasing the number of cells in the S phase, but the maximal increase in cell number was obtained in the presence of antioxidants (Figure 2B and Additional file 2: Figure S2). As shown in Additional file 3: Figure S3, NAC alone (ImF-Nac), AsA2P alone (ImF-AsA2P), or NAC+AsA2P (ImF-A) all resulted in higher cell proliferation as compared to the control medium (ImF). We also found that exposure to antioxidants and FGF-2 stimulated the proliferation of MSCs obtained from adult human liposuction aspirates (Figure 2B), bone marrow and Wharton's jelly (Additional file 4: Figure S4).

Antioxidants [32] and hypoxia [33] are believed to decrease intracellular ROS levels, and intracellular ROS play key roles in regulating cell adhesion and cell proliferation [34]. Hypoxia is also known to affect the secretion of several growth factors [13,33], such as insulin-like growth factor, VEGF, hepatocyte growth factor, HIF, and FGF-2, which have been shown to accelerate the expansion of BMMSCs [11]. However, HIF1A can enhance hepatocyte growth factor and FGF-2 secretion from MSCs [33], and TGF-β1 can inhibit the proliferation of human prostatic epithelial cells by upregulating the levels of p15, p21, and p27 and by arresting the cell cycle in the G1 phase [35]. Recently, Tsai et al. reported that MSCs cultured under hypoxia can increase expansion, bypass cellular senescence, and maintain stem cell properties by increasing the proportion of cells in the S phase and suppressing E2A-p21 expression through the HIF-TWIST pathway [36]. In this study, *HIF1A* and *VEGF* were significantly upregulated in the ImF, ImF-H, and ImF-A groups, whereas *TGF-β1* was significantly downregulated (Figure 4B and Additional file 5: Figure S5). Therefore, it is possible that the ImF-A-treated or ImF-H-treated cells switched on their cell cycle program through autocrine and paracrine effects via a similar pathway. This hypothesis is further supported by the results of our western blot analysis (Figure 3C) that showed similar cell cycle-associated regulator profiles in Figure 3C between the ImF-A and ImF-H treatments. Most interestingly, addition of antioxidants also suppressed the cellular reactive oxygen species (ROS) and restored cell proliferation of ADMSCs under hyperoxia (37.5% pO_2_, Additional file 6: Figure S6A-6C). The suppression of ROS and restoration of cell growth by antioxidants were associated with the restoration of p21 and CDK2 in western blot analysis (Additional file 6: Figure S6D).

Some studies have found that the expression of CD90 and CD105 was lower in hypoxia-expanded MSCs than in normoxia-expanded MSCs [37,38]. In this study, we observed that ADMSCs are positive for the cell-surface markers CD13, CD29, CD44, CD73, CD90, CD105, and B2M and negative for CD14, CD34, CD45, CD235a, and HLA-DR; however, the degree of expression of some of these markers varied depending on the culture conditions. We also found that both antioxidants and hypoxia decreased the expression of CD29 (integrin β1; fibronectin receptor), CD90 (Thy-1; cell-matrix interactions), CD105 (endoglin; matrix protein), and B2M (a component of MHC class I molecules). Hypoxia promotes MSCs migration [13,39] and their mobilization into the peripheral blood [40]. However, the decreased expression of CD29, CD90, and CD105 by these cells in the ImF-H and ImF-A groups may be associated with changes in cell migration or cell-matrix interactions. This hypothesis is supported by our observation that cell surface antigen profiles were similar in the ImF-A and ImF-H groups.

We also found that antioxidants affect the expression levels of stemness genes (Figure 4) and the differentiation potential (Figure 6) of ADMSCs. The expression of *Oct4*, *Nanog*, *SOX2*, and *CXCR4* was higher under hypoxic condition than under normoxic conditions (Additional file 7: Figure S7), similar to the results of previous studies [13-15,31]. In our experiments, stemness genes were significantly upregulated when FGF-2 was added to the medium, but the expression levels of *Nanog*, *Oct4*, and *CXCR4* were higher in the ImF-A group than in the ImF-H group. Hypoxia can enhance the osteogenic potential of BMMSCs, although it also inhibits their adipogenic potential [12,13]. When the accumulation of intracellular ROS in BMMSCs was inhibited by NAC, the adipogenic differentiation of BMMSCs was also inhibited [32]. FGF-2 also enhances the osteogenic [11], chondrogenic [26], and adipogenic potential [41] of ADMSCs. In the present study, we also observed differences in the differentiation potential of ADMSCs among the treatment groups. ADMSCs exposed to FGF-2 had relatively greater differentiation potential; however, this potential was greatly enhanced in cells treated with ImF-H or ImF-A.

## Conclusions

We infer that antioxidants can modulate the cell cycle progression of ADMSCs by downregulating CDK and CDK4 inhibitors and upregulating CDK2, CDK4, and CDC2 expression. In the presence of antioxidants and FGF-2, ADMSCs rapidly proliferate and retain their stem cell properties, and their osteogenic and adipogenic potentials are enhanced. The mechanisms by which antioxidants induce cell proliferation are possibly the same as those involved in hypoxia, but the *in vitro* culture environment of antioxidant-supplemented medium under normoxic conditions may be more stable than hypoxic conditions. Thus, we propose that a combination of antioxidants and FGF-2 under normoxic conditions may provide an easy and rapid method for *in vitro* expansion of both ADMSCs and BMMSCs. Further development of this method for culturing MSCs in bioreactors may provide more functional MSCs for cell therapy and reduce patient waiting time without compromising the capacity of these cells to differentiate.

## Abbreviations

ADMSCs: Adipose-derived mesenchymal stem cells; MSCs: Mesenchymal stem cells; ROS: Reactive oxygen species; FGF-2: Fibroblast growth factor-2; CDK: Cyclin-dependent kinase; IMDM: Iscove’s modified Dulbecco’s medium; FBS: Fetal bovine serum; B2M: β_2_ microglobulin; CDK4: Cyclin-dependent kinase 4; CDK6: Cyclin-dependent kinase 6; CDC2: Cell division control protein 2; p21: Cyclin-dependent kinase inhibitor 1A; p27: Cyclin-dependent kinase inhibitor 1B; Oct4: Octamer-binding transcription factor 4; SOX2: Sex-determining region Y-box 2; CXCR4: C-X-C chemokine receptor type 4; HIF1A: Hypoxia inducible factor 1 alpha subunit; VEGF: Vascular endothelial growth factor; TGF-β1: Transforming growth factor beta 1; cbfa1: Core-binding factor subunit alpha-1; OC: Osteocalcin; PPARr: Proliferator-activated receptor γ; C/EBPα: CCAAT⁄enhancer-binding protein α; ACAN: Aggrecan; COL2A1: Collagen type II, alpha 1; RT-qPCR: Quantitative reverse transcription PCR; Im: IMDM with 10% FBS; ImF: Im with 10 ng/mL FGF-2; ImF-H: ImF under 5% pO_2_ environment; ImF-A: ImF with 2 mM *N*-acetyl-l-cysteine and 0.2 mM ascorbic acid-2-phosphate.

## Competing interests

The authors declare that they have no competing interests.

## Authors’ contributions

LYS and CYP contributed equally to this study. All authors have read and approved the final version of the manuscript.

## Supplementary Material

Additional file 1: Figure S1 Effects of FGF-2, antioxidants and different oxygen partial pressure on the expressions of CD105 in ADMSCs. The proliferated ADMSCs were labeled with EdU (5-ethynyl-2′-deoxyuridine) by cultured in medium with 5 μM EdU overnight as recommended by the manufacturer (Click-iT® EdU Alexa Fluor® 488 Flow Cytometry Assay Kit, Molecular Probes). CD105 expression and the percentage of EdU-labeled ADMSCs were measured by flow cytometry. In the negative control experiment, the ADMSCs were cultured under the same conditions without EdU. Density level threshold, 50 cells, and density levels of 1–5 indicated by color (blue to red). Click here for file

Additional file 2: Figure S2 Effects of antioxidants on ADMSCs proliferation under normoxic or hypoxic conditions. ADMSCs were cultured in Im, Im-H (hypoxia), and Im-A (containing antioxidants: NAC and AsA2P) at an initial cell density of 3,000 cells/cm^2^, respectively. Medium was changed every 3 days, starting at day 3. Each bar represents the mean value ± SD (n = 3). * *P* < 0.05. Click here for file

Additional file 3: Figure S3 Effects of different antioxidants on ADMSCs (A) proliferation and (B) cell cycle-regulated proteins. Click here for file

Additional file 4: Figure S4 Effects of antioxidants on different type of MSCs under normoxic or hypoxic conditions. **(A)** Adipose-derived mesenchymal stem cells (ADMSCs). **(B)** Bone marrow mesenchymal stem cells (BMMSCs). **(C)** Wharton’s jelly stem cells (WJSCs). Medium was changed every 3 days, starting at day 3. Each bar represents the mean value ± SD (n = 3). * *P* < 0.05. Click here for file

Additional file 5: Figure S5 Effects of antioxidants and hypoxic condition on HIF-1α expression in ADMSCs. Expression of *β-actin* served as the internal control. The relative expressions were expressed as fold induction as compared to ImF. Click here for file

Additional file 6: Figure S6 Effects of antioxidants and different oxygen partial pressure on cellular H_2_O_2_ and cell proliferation of ADMSCs. (A) ROS level of ADMSCs cultured under normoxia with/without antioxidant or under hypoxia (5% pO_2_) on Day 5. (B) ROS level of ADMSCs cultured under normoxia or under hyperoxia (37.5% pO_2_) with/without antioxidant on Day 5. (C) Proliferation of ADMSCs cultured under normoxia with/without antioxidant (ImF / ImF-A) or under hyperoxia with/without antioxidant (ImF(37.5% pO_2_) / ImF-A(37.5% pO_2_)). (D) Western blot analysis of p21, CDK2 expressions on Day 5. Click here for file

Additional file 7: Figure S7 Effects of hypoxia or antioxidants on stemness genes expression in ADMSCs without FGF-2. Expression of *β-actin* served as the internal control. The expression values were normalized to the corresponding gene measured in cells cultured in medium alone. Each bar represents the mean value ± SD (n = 3). * *P* < 0.05. Click here for file
