# Supplementary figures and images for: Antioxidants cause rapid expansion of human adipose-derived mesenchymal stem cells via CDK and CDK inhibitor regulation
Source: J Biomed Sci. 2013 Aug 1;20(1):53. doi: 10.1186/1423-0127-20-53 (PMC3751058; doi:10.1186/1423-0127-20-53)

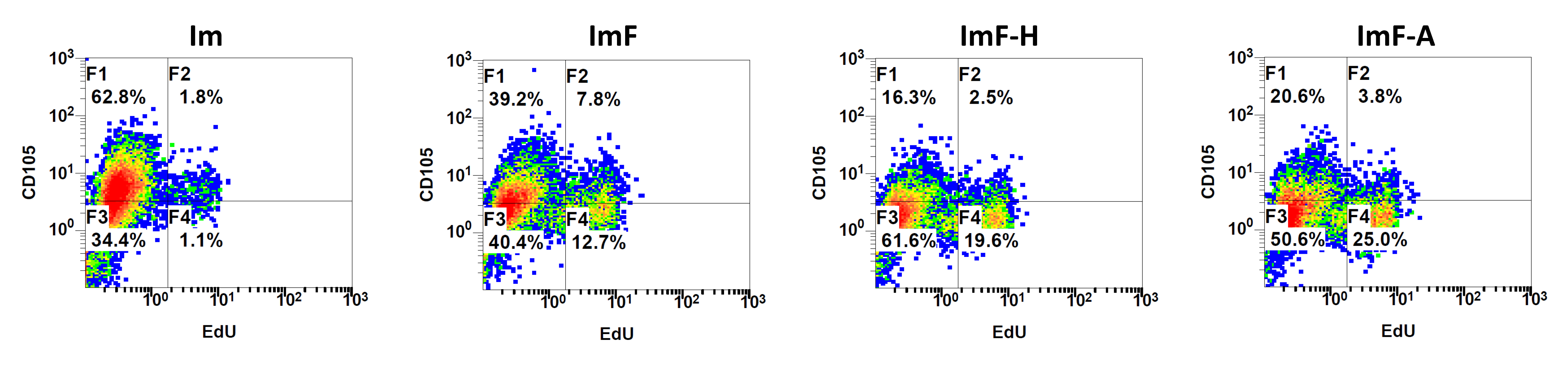

Supplement: Additional file 1: Figure S1 — Effects of FGF-2, antioxidants and different oxygen partial pressure on the expressions of CD105 in ADMSCs. The proliferated ADMSCs were labeled with EdU (5-ethynyl-2′-deoxyuridine) by cultured in medium with 5 μM EdU overnight as recommended by the manufacturer (Click-iT® EdU Alexa Fluor® 488 Flow Cytometry Assay Kit, Molecular Probes). CD105 expression and the percentage of EdU-labeled ADMSCs were measured by flow cytometry. In the negative control experiment, the ADMSCs were cultured under the same conditions without EdU. Density level threshold, 50 cells, and density levels of 1–5 indicated by color (blue to red). [file 1423-0127-20-53-S1.tif]

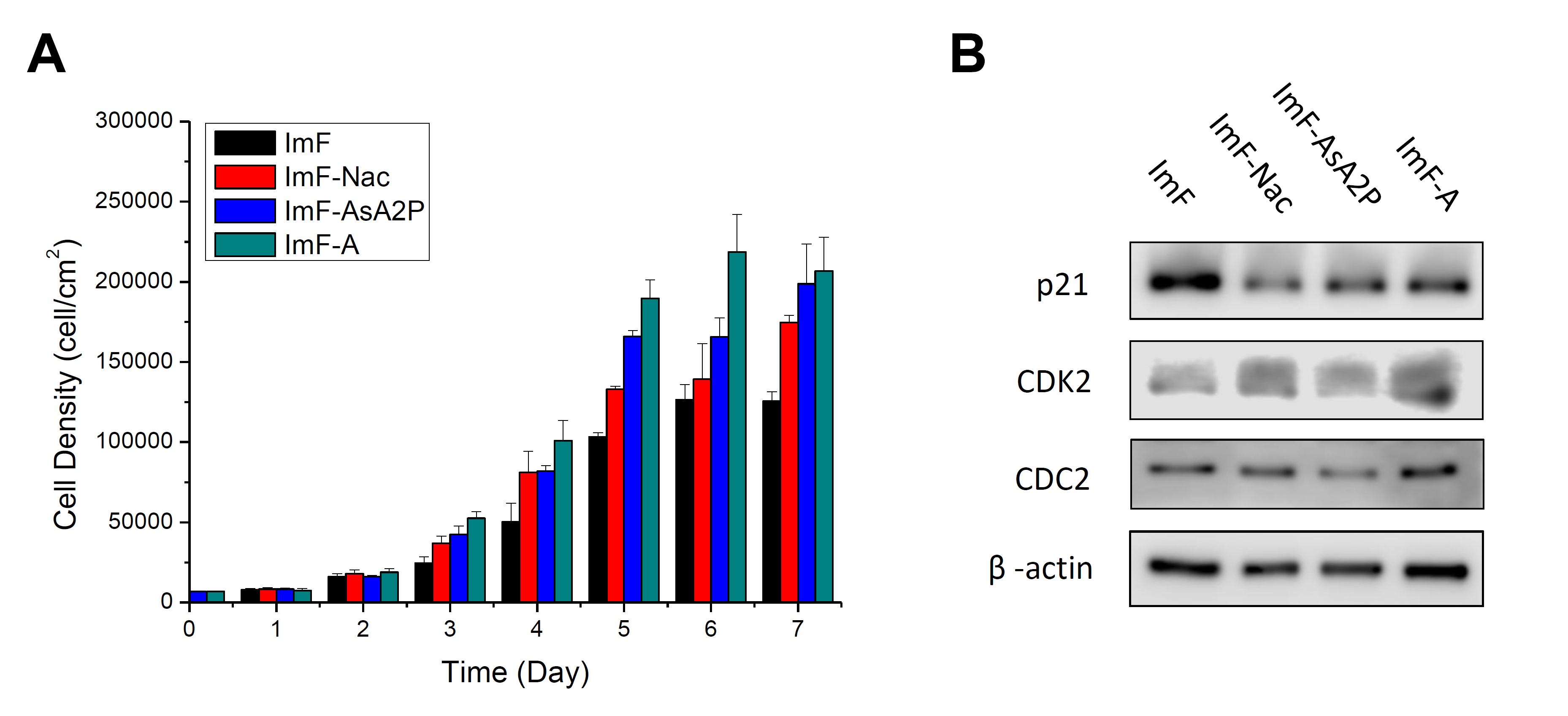

Supplement: Additional file 3: Figure S3 — Effects of different antioxidants on ADMSCs (A) proliferation and (B) cell cycle-regulated proteins. [file 1423-0127-20-53-S3.tif]

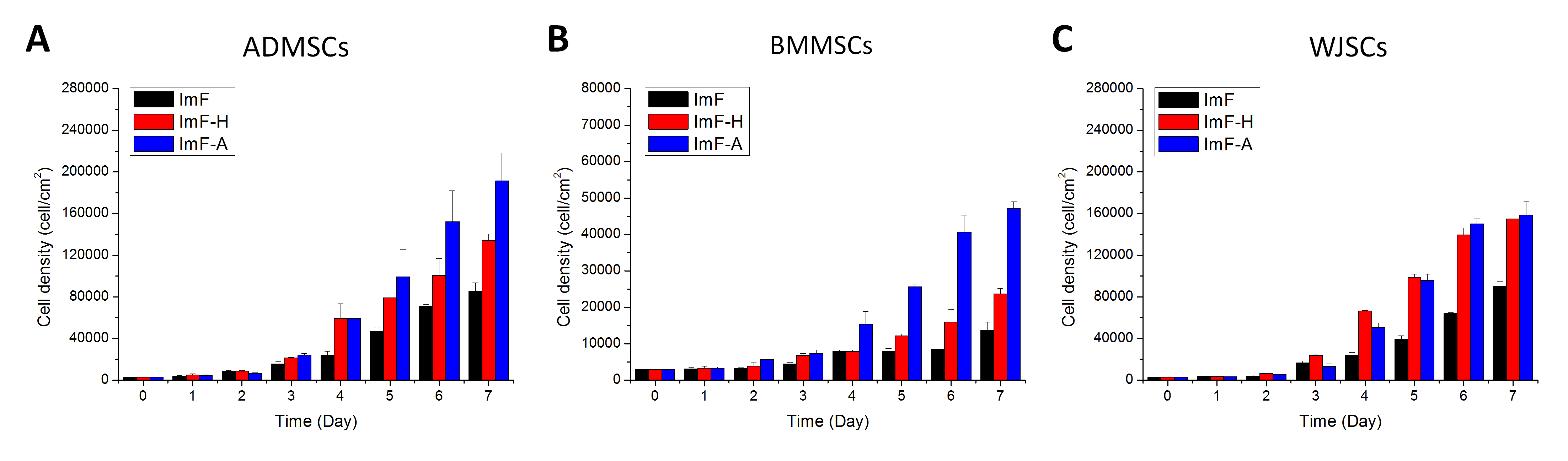

Supplement: Additional file 4: Figure S4 — Effects of antioxidants on different type of MSCs under normoxic or hypoxic conditions. (A) Adipose-derived mesenchymal stem cells (ADMSCs). (B) Bone marrow mesenchymal stem cells (BMMSCs). (C) Wharton’s jelly stem cells (WJSCs). Medium was changed every 3 days, starting at day 3. Each bar represents the mean value ± SD (n = 3). * P < 0.05. [file 1423-0127-20-53-S4.tif]

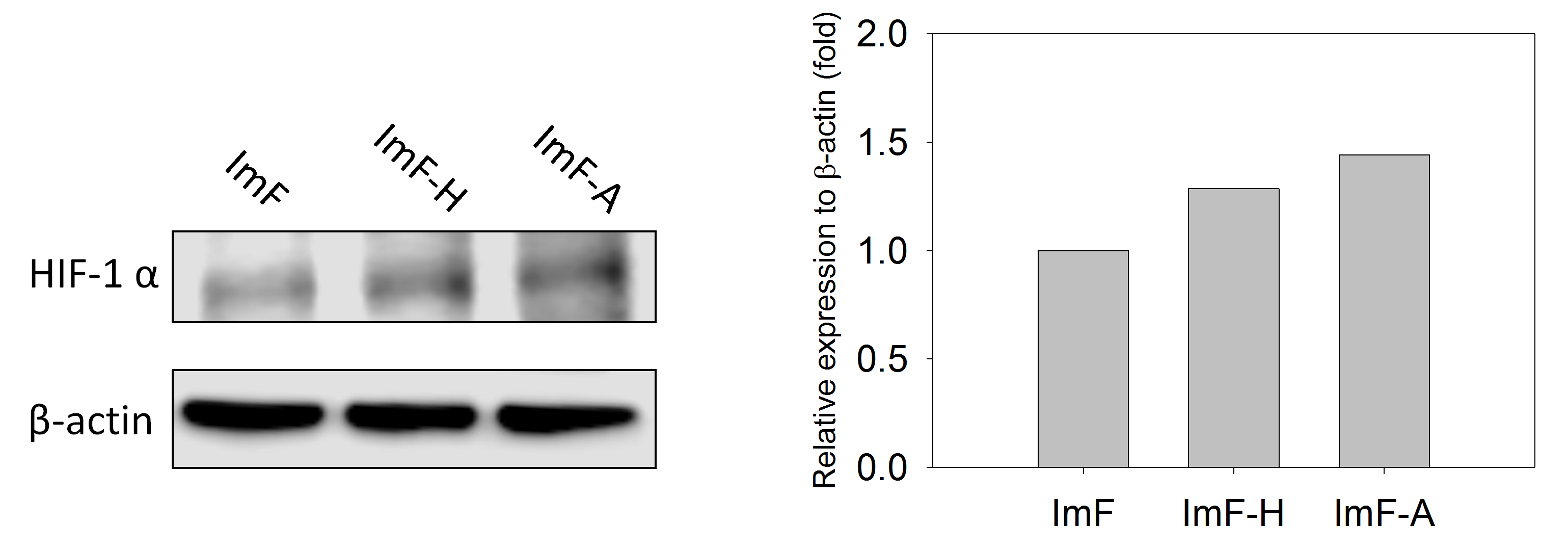

Supplement: Additional file 5: Figure S5 — Effects of antioxidants and hypoxic condition on HIF-1α expression in ADMSCs. Expression of β-actin served as the internal control. The relative expressions were expressed as fold induction as compared to ImF. [file 1423-0127-20-53-S5.tif]

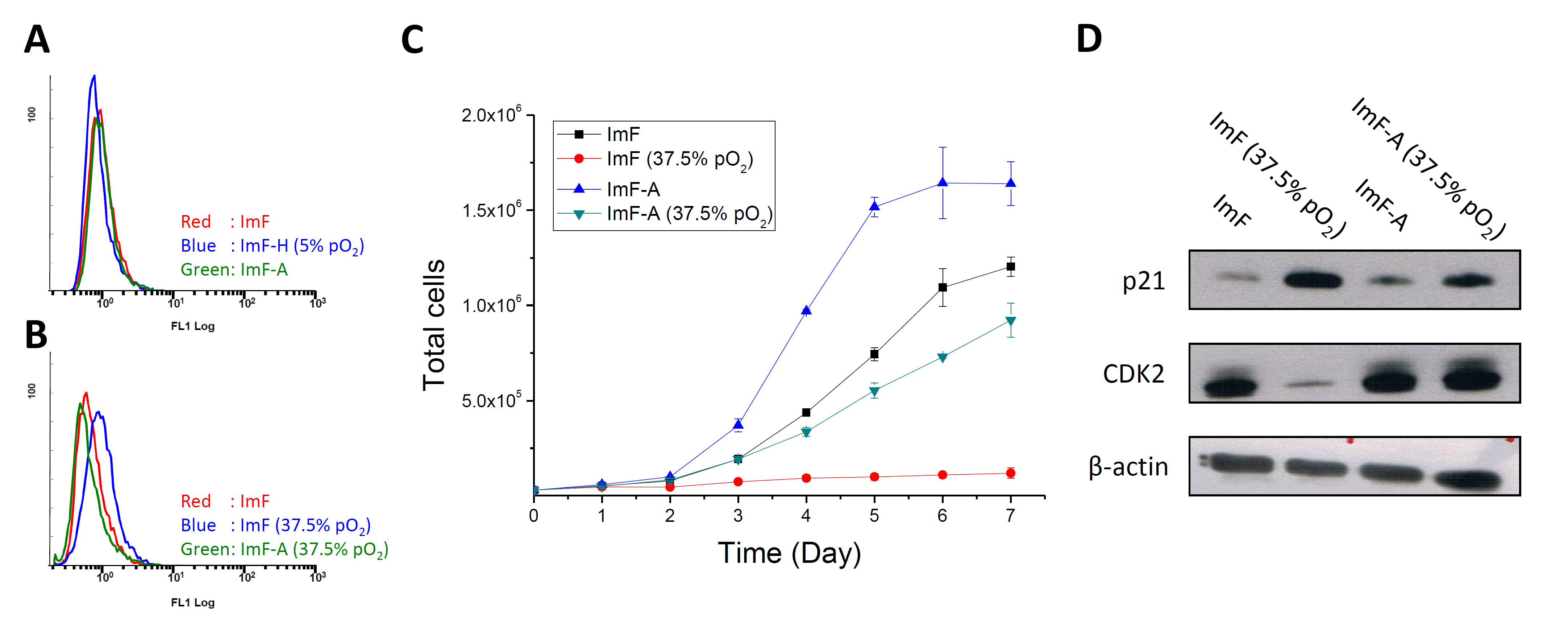

Supplement: Additional file 6: Figure S6 — Effects of antioxidants and different oxygen partial pressure on cellular H2O2 and cell proliferation of ADMSCs. (A) ROS level of ADMSCs cultured under normoxia with/without antioxidant or under hypoxia (5% pO2) on Day 5. (B) ROS level of ADMSCs cultured under normoxia or under hyperoxia (37.5% pO2) with/without antioxidant on Day 5. (C) Proliferation of ADMSCs cultured under normoxia with/without antioxidant (ImF / ImF-A) or under hyperoxia with/without antioxidant (ImF(37.5% pO2) / ImF-A(37.5% pO2)). (D) Western blot analysis of p21, CDK2 expressions on Day 5. [file 1423-0127-20-53-S6.tif]

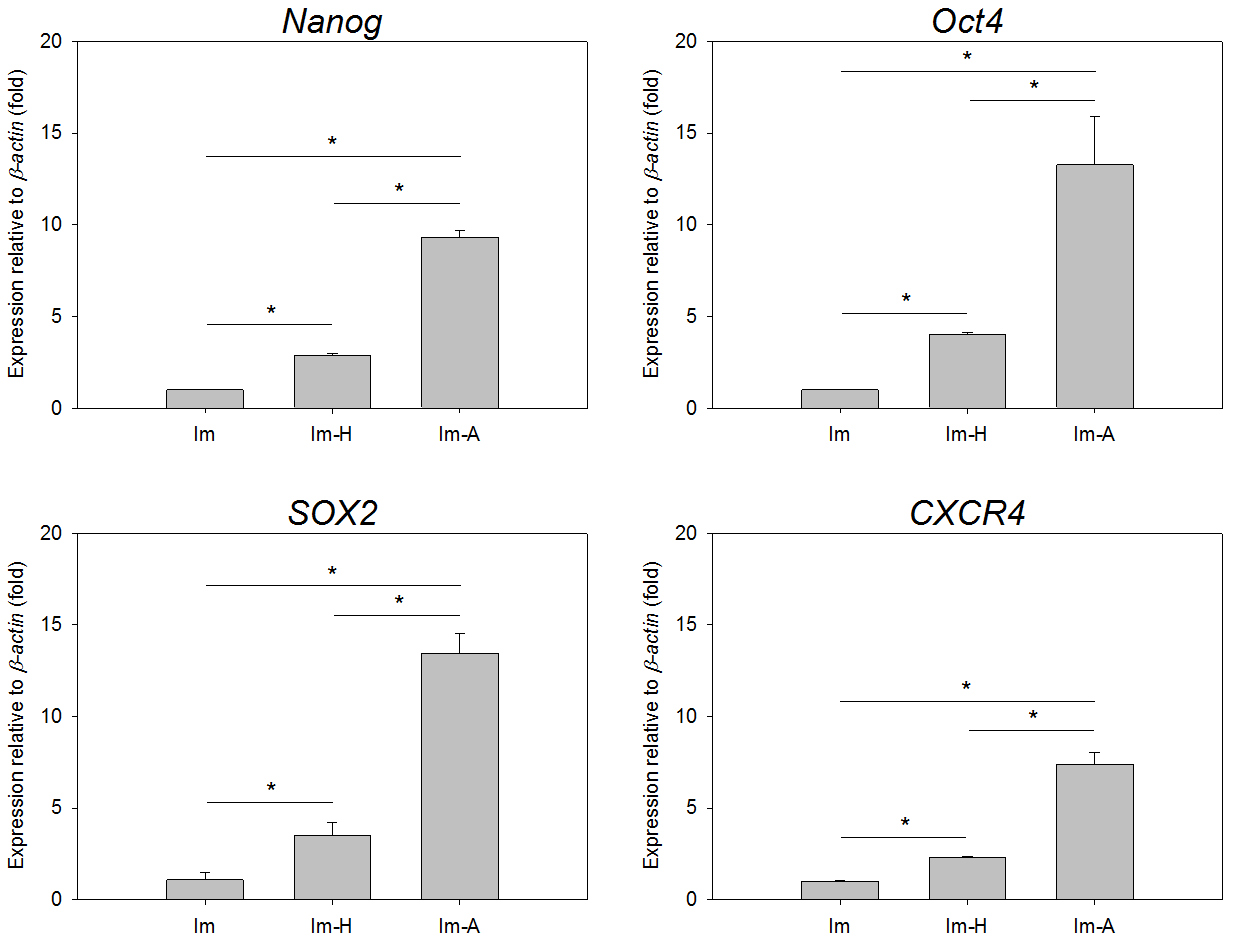

Supplement: Additional file 7: Figure S7 — Effects of hypoxia or antioxidants on stemness genes expression in ADMSCs without FGF-2. Expression of β-actin served as the internal control. The expression values were normalized to the corresponding gene measured in cells cultured in medium alone. Each bar represents the mean value ± SD (n = 3). * P < 0.05. [file 1423-0127-20-53-S7.tif]
